# Evaluation of a Smartphone-Based Training Strategy Among Health Care Workers Screening for Cervical Cancer in Northern Tanzania: The Kilimanjaro Method

**DOI:** 10.1200/JGO.2015.001768

**Published:** 2016-05-04

**Authors:** Karen E. Yeates, Jessica Sleeth, Wilma Hopman, Ophira Ginsburg, Katharine Heus, Linda Andrews, Mary Rose Giattas, Safina Yuma, Godwin Macheku, Aziz Msuya, Olola Oneko

**Affiliations:** **Karen E. Yeates**, **Jessica Sleeth**, and **Wilma Hopman**, Queen’s University, Kingston; **Ophira Ginsburg**, University of Toronto, Toronto, Ontario, Canada; **Katharine Heus**, Pamoja Tunaweza Women’s Centre; **Linda Andrews**, International Center for AIDS Care and Treatment Programs Tanzania; **Mary Rose Giattas**, Johns Hopkins Program for International Education in Gynecology and Obstetrics; **Safina Yuma**, Tanzanian Ministry of Health and Social Welfare, Dar es Salaam; **Godwin Macheku**, Mawenzi Government Hospital; **Aziz Msuya**, Arumeru District Hospital, Arumeru; and **Olola Oneko**, Kilimanjaro Christian Medical College, Moshi, Tanzania.

## Abstract

**Purpose:**

Almost nine of 10 deaths resulting from cervical cancer occur in low-income countries. Visual inspection under acetic acid (VIA) is an evidence-based, cost-effective approach to cervical cancer screening (CCS), but challenges to effective implementation include health provider training costs, provider turnover, and skills retention. We hypothesized that a smartphone camera and use of cervical image transfer for real-time mentorship by experts located distantly across a closed user group through a commercially available smartphone application would be both feasible and effective in enhancing VIA skills among CCS providers in Tanzania.

**Methods:**

We trained five nonphysician providers in semirural Tanzania to perform VIA enhanced by smartphone cervicography with real-time trainee support from regional experts. Deidentified images were sent through a free smartphone application on the available mobile telephone networks. Our primary outcomes were feasibility of using a smartphone camera to perform smartphone-enhanced VIA and level of agreement in diagnosis between the trainee and expert reviewer over time.

**Results:**

Trainees screened 1,072 eligible women using our methodology. Within 1 month of training, the agreement rate between trainees and expert reviewers was 96.8%. Providers received a response from expert reviewers within 1 to 5 minutes 48.4% of the time, and more than 60% of the time, feedback was provided by regional expert reviewers in less than 10 minutes.

**Conclusion:**

Our method was found to be feasible and effective in increasing health care workers’ skills and accuracy. This method holds promise for improved quality of VIA-based CCS programs among health care providers in low-income countries.

## INTRODUCTION

Almost nine (87%) of 10 deaths resulting from cervical cancer occur in low-income countries.^1^ In 2012, cervical cancer was the fourth most common cancer among women worldwide, with an estimated 528,000 new cases and 266,000 deaths, accounting for 7.5% of all female deaths resulting from cancer. Cervical cancer mortality is high in sub-Saharan Africa (SSA), with cervical cancer age-standardized mortality rates as high as 56.4 in Zimbabwe, 54 in Tanzania, and 75.9 in Malawi per 100,000.^2^ The lack of available comprehensive screening programs for cervical cancer in SSA helps explain why more than 50% of women with cervical cancer receive their diagnosis at an advanced disease stage, when curable treatment options are limited and survival rates are poor.^3^ In a recent study from the Kilimanjaro region of Tanzania, 82% of women reported they had knowledge of cervical cancer, although only 6% had ever been screened for cervical cancer, which is similar to the WHO estimate of 5% for 5-year cervical cancer screening (CCS) prevalence in developing countries.^4^

Numerous studies have shown that visual inspection of the cervix with the naked eye after application of 3% to 5% acetic acid solution (VIA) is the best available CCS approach in low-income countries, and in 2013, the WHO endorsed the use of VIA and cryotherapy-based screen-and-treat programs in countries without existing comprehensive cervical cancer services.^5^ VIA can be performed by nurses and other health practitioners, is low in cost, and saves lives when treatment with cryotherapy is offered on site for small to moderate lesions. For larger lesions, the loop electrosurgical excision procedure (LEEP) is offered through properly linked referral services.^6-9^

In Tanzania, the Ministry of Health and Social Welfare (MOHSW) previously adopted VIA as a national screening strategy, and in May 2011, the Tanzania Service Delivery Guidelines for Cervical Cancer Prevention and Control were printed and distributed. To adequately train CCS providers, the MOHSW has established a 6-day competency-based training program for health care workers including nurses and clinicians. Because it takes experience to access the cervix and perform VIA, the MOHSW recommends that trainees who are not qualified after training be mentored by an experienced provider or regional or district-based CCS trainer until they have accurately screened 50 clients and identified and treated at least five precancerous lesions accurately. To help maintain quality service, there is to be ongoing quarterly technical supervision by a district CCS trainer. The guidelines also advocate for a monthly program of supervision by the district reproductive child health coordinator, which has not been feasible to implement in Tanzania because of trainer shortages and lack of financial resources. Ongoing challenges in training are a lack of post-training mentorship and lack of ongoing technical supportive supervision of CCS providers combined with the high turnover of trained providers. In programs where cryotherapy is not offered at the time of screening, loss to follow-up can be high, highlighting that screening is only effective if offered with treatment at the same visit^10-12^ and further emphasizing the importance of skilled providers.

One adjunct to improving training for and quality of VIA is the use of a digital camera linked to a television or computer monitor. This method is known as digital cervicography and consists of provision of VIA followed by an additional step whereby a digital camera is used to transmit a photographic image of the cervix to a television screen or computer monitor that can be reviewed at higher clarity and resolution than is available with the naked eye.^13,14^ This method has been in use in Zambia since 2006, with well-established programs to improve nurse training in CCS and treatment.^8,15^ The Zambian program has incorporated Internet transfer of digital cervical photographic images for review and mentorship by experts at a coordinating site.^7,16^ This method allows for ongoing oversight or training of new sites but requires a reliable Internet connection, digital camera, laptop and/or television monitor, and an electricity source, making it less feasible in many low-income countries with limited health resources. In Tanzania, there is one site located in the northern area of the country that has an active digital cervicography program. This program has never successfully been scaled to other sites because of a lack of resources.

The increasing availability and affordability of smartphones and the widespread availability of reliable mobile telephone networks in Tanzania have provided a promising solution to the challenges of digital cervicography. With the knowledge that one of the biggest challenges in creating successful VIA programs was ensuring CCS providers had acquired the necessary clinical skills in VIA, health provider confidence, and quality of screening over time, we hypothesized that smartphone cameras could provide a viable option to improve mentorship, oversight, and measureable quality assurance for newly trained and existing CCS providers. At study inception and at the time of writing, we were unable to find evidence of a smartphone-based cervicography program that has been tested within an existing VIA program in a low-income country. We sought to evaluate the effectiveness of a smartphone-based cervicography and text message (image transfer) platform to enhance VIA training, quality, and accuracy through real-time mentorship and training of health care workers providing CCS in semirural Tanzania. As our method differs from traditional cervicography and to more clearly describe this technique, we describe it as smartphone-enhanced VIA (SEVIA).

## METHODS

### Study Population

The study took place in the northern zone of Tanzania. The only functioning digital cervicography program in Tanzania is situated at the Reproductive Health Center located at Kilimanjaro Christian Medical Center (KCMC) in Moshi, Kilimanjaro. One local cervical cancer prevention expert who was the founder of the KCMC digital cervicography program participated throughout the inception and implementation of the study (O.O.). Two other digital cervicography experts (one physician and one nurse) also participated in development and implementation of the training program and provided expert mentorship of trainees at the participating satellite site through sharing of images through our SEVIA technique. Before the initiation of the study, we field-tested different smartphones for resolution and ease of use and selected the iPhone 5S (Apple, Cupertino, CA) for its clarity and resolution of photographic images.

### Study Location

We selected a geographically distinct site that was participating in the Tanzanian Ministry of Health VIA-based CCS program. Arumeru District Hospital is located 100 km from Moshi in Meru District, is a semirural health facility that serves a population of almost 1 million people, and provides a variety of hospital-based services including maternal and child health. At the time of engagement in the research program, the hospital was providing opportunistic VIA to women who presented to the health center for screening.

### Study Trainees and Screening Participants

The trainees consisted of three nurses and two assistant medical officers. In Tanzania, these types of health care workers provide much of the day-to-day health care to patients in the health system. All of the trainees had undergone a VIA training program in the past and had (on average) 2 years of experience but had not had their VIA skills re-evaluated until our training program, conducted as part of this study. After verbally agreeing to participate in the study, these five CCS providers underwent the MOHSW 6-day competency-based training program. Written informed consent was not obtained, because these individuals were receiving training to enhance their usual day-to-day work activities, and the evaluation component was related only to the accuracy of their skills in a blinded fashion. After the VIA training program, they underwent training to learn how to use the smartphone camera and how to acquire good-quality cervical images and save and transfer images, as well as training on the study protocol to maintain confidentiality and consistency to troubleshoot potential problems with the technology. This was to ensure that the providers had the necessary knowledge and skills to perform VIA and smartphone cervicography. In addition to the trainees, study participants also included female patients who were deemed eligible for screening if they were sexually active women age 25 to 49 years. Women who had conditions that would interfere with clear visualization of the cervix (eg, heavy menses, severe cervicitis, or surgical removal of the cervix) were excluded, as were those who had a known history of cervical, endometrial, vulvar, or ovarian cancer.

In the event that a prolonged power outage occurred and to ensure they were able to charge the smartphones during mobile clinics located off site, we equipped the trainees with a solar-powered device that was a combination of a light-emitting diode light source and mobile telephone battery charger. Using this device, the smartphones could remain charged and the screening team would always have an available light source to perform the screening in a variety of clinic settings (solar lanterns were provided by StarEcoworks, Calgary, Alberta, Canada). All CCS staff who participated in the study and the expert reviewers were employed by a government institution.

From the period of June 1, 2014, to March 31, 2015, the newly trained Arumeru CCS team performed VIA supplemented by SEVIA as part of the study protocol. All female patients visiting Arumeru Hospital for either CCS or other care through the maternal and child health clinic were offered participation in the research program. All female participants provided informed consent. If they were able to read the consent form (in Kiswahili), the form was used to guide the discussion, and they were able to sign the form if they consented to participation. Those who declined to have their cervical images taken and used in the study protocol were offered both VIA and smartphone cervicography (if desired), but their data were not included in the study results. Only one woman declined to participate in the research program. To support the MOHSW integrated approach in reproductive maternal and child health programming, all participants who did not know their HIV status were offered HIV testing and counseling as per MOHSW guidelines and appropriate referral (if positive) before VIA and SEVIA.

### SEVIA Protocol

**Fig 1:**
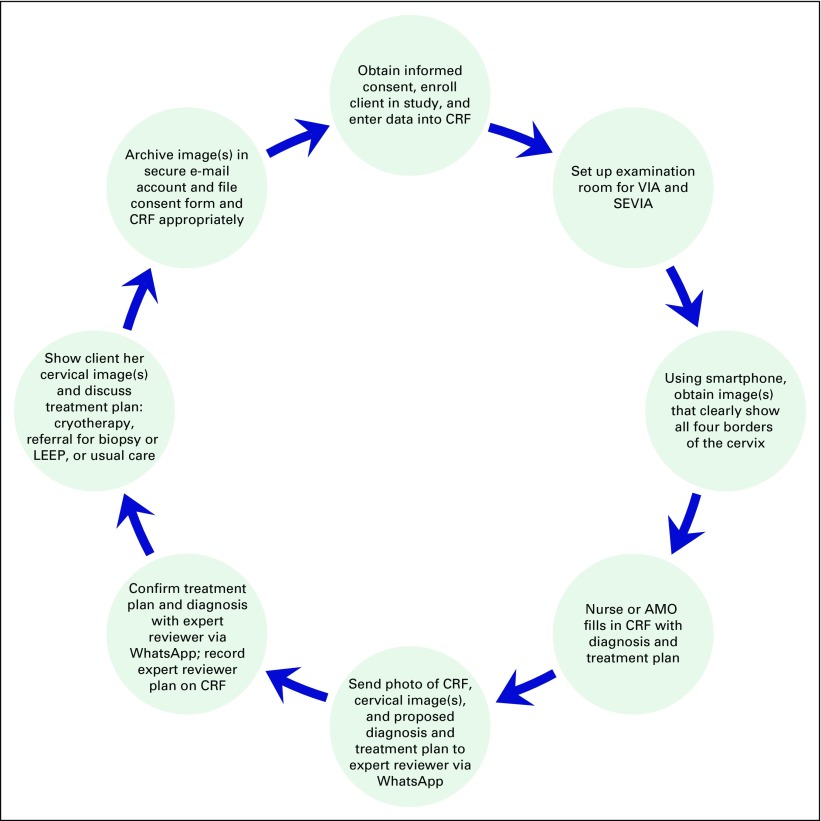
Flowchart of visual inspection under acetic acid (VIA) and cervical image ascertainment and smartphone image transfer methodology. AMO, assistant medical officer; CRF, case report form; LEEP, loop electrosurgical excision procedure; SEVIA, smartphone-enhanced cervicography.

Participants received standardized counseling about the VIA and SEVIA procedures. After VIA was performed (and results recorded), the health provider immediately performed cervicography with the smartphone camera. Depending on a woman’s size and parity, between one and four images of the cervix were obtained by dividing the cervix into four quadrants. The image collection, documentation procedure on a case report form, and image transfer for mentorship protocol are presented in Fig 1. After screening was completed, the result was then communicated to the participant through a counseling session that typically lasted 2 minutes, and she was shown her cervical images to enhance knowledge regarding her own body and the risks of cervical cancer. During this counseling session, follow-up and treatment plans were devised as per standard MOHSW guidelines, including offering immediate cryotherapy, referral for LEEP, referral for biopsy, or further staging for more-advanced cervical cancer lesions. All deidentified study images and data were stored on a secure online platform.

After 6 months, the team added a once-per-week mobile component by expanding the identical protocol and program to rural community-based sites where women have limited access to CCS or face barriers resulting from cost of travel for screening. These results were also included in this analysis.

### Ethical Considerations

The study was approved by the institutional review boards of the KCMC, Queen’s University (Kingston, Ontario Canada), and the National Institute for Medical Research Tanzania.

### Statistical Analysis

Data were entered into an Excel file and imported into IBM SPSS (version 22.0) for Windows (Armonk, NY) for statistical analysis. Data were initially examined descriptively, including frequencies and percentages for categorical data and means and standard deviations for continuous data (age and parity). Those with and without a disagreement between the CCS provider and expert were compared using χ^2^ tests for categorical data (Pearson’s or Fisher’s as appropriate) and independent-samples *t* tests for continuous data. Because parity was not normally distributed, it was also tested using the Mann-Whitney U test statistic.

## RESULTS

During the study period, a total of 1,072 women were screened with VIA plus SEVIA by the team of five CCS providers. In Table 1, we list sociodemographic characteristics, parity, and HIV status of the women screened. Most women had completed only primary school and were married; 9.6% (103) were HIV positive.

**Table 1:**
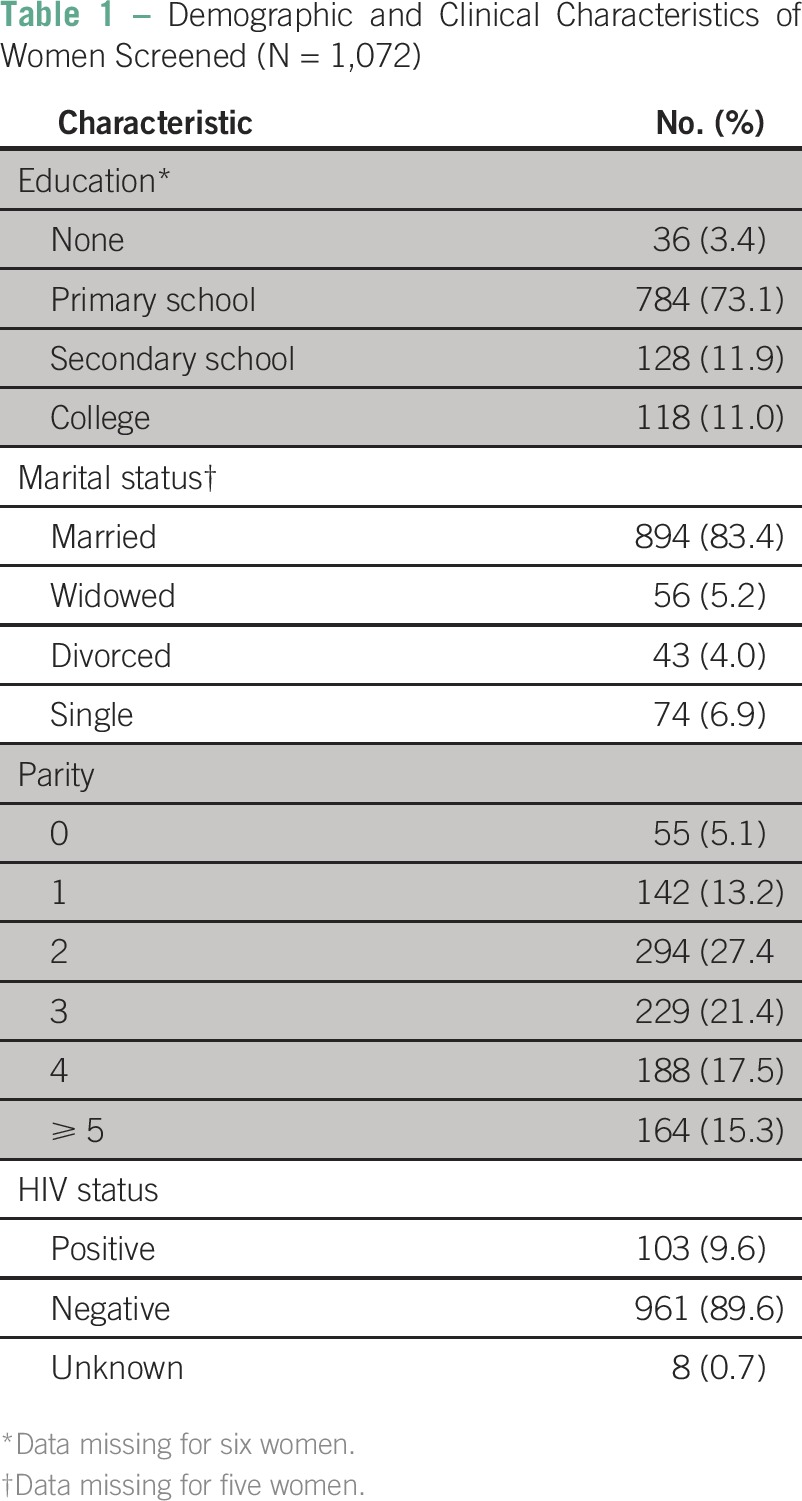
Demographic and Clinical Characteristics of Women Screened (N = 1,072)

Table 2 summarizes the level of agreement between the CCS providers versus the experts in addition to operating characteristics such as the time it took to complete the VIA plus smartphone cervicography and to receive expert feedback. Overall, there was 96.8% agreement between the CCS providers and expert reviewers. This occurred after 1 month of starting the program of expert review of and feedback on the screening images through the closed user group text message platform. A total of 92.3% of the cervical images were collected between 1 and 5 minutes. The time to receive expert feedback was longer, with 48.4% of the feedback obtained between 1 and 5 minutes and 38.6% requiring more than 10 minutes. Typical reasons for delays in expert feedback were documented (data not shown); these including mobile telephone network outages (15%), expert reviewer busy in the operating theater (10%), on the telephone (8%), or driving (7%), and other reasons. In Table 2, we also list screening outcomes for VIA and SEVIA. With VIA screening alone, the overall VIA positivity rate was 10.6% (n = 114 of 1,069), and 0.2% (n = 2) were suggestive of cancer. When SEVIA was added, results were almost the same; the overall VIA positivity rate was 10.9% (n = 117 of 1,070), and 0.4% (n = 4) were suggestive of cancer. Table 3 lists VIA and SEVIA results. In our study, 10.6% of women were VIA positive, and 88.9% were negative, with 0.2% suggestive of cancer. The SEVIA rates were 10.9% positive and 88.5% negative, with 0.4% suggestive of cancer.

**Table 2:**
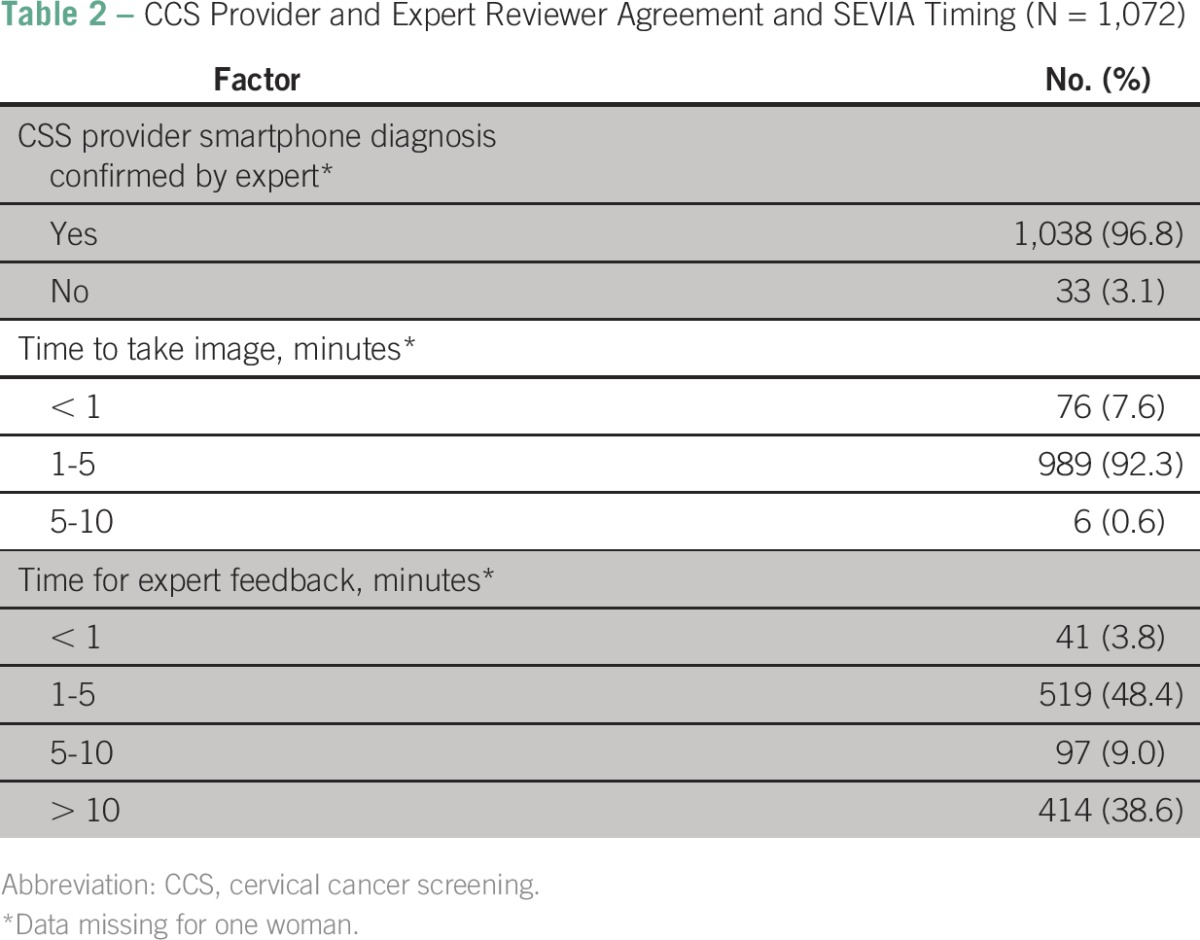
CCS Provider and Expert Reviewer Agreement and SEVIA Timing (N = 1,072)

**Table 3:**
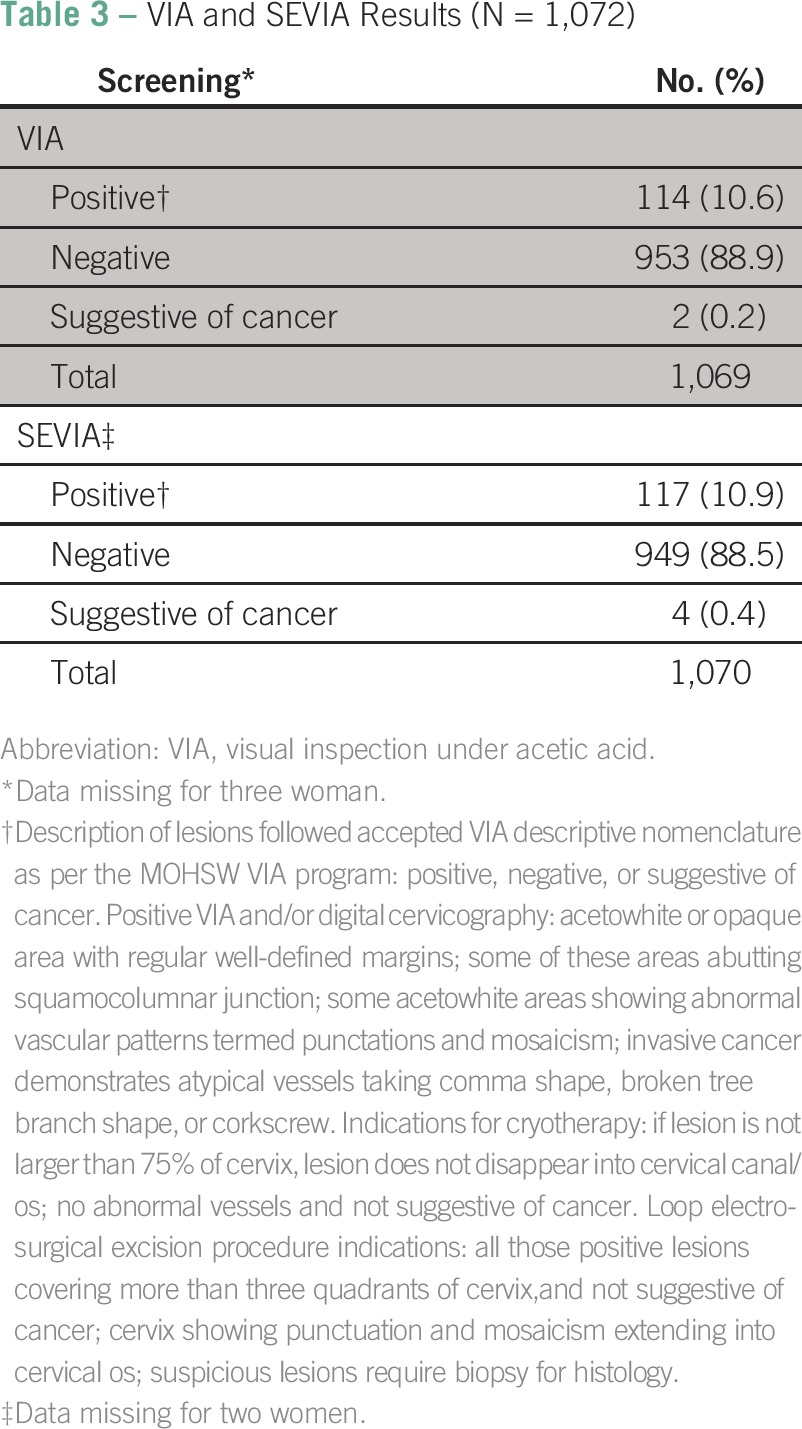
VIA and SEVIA Results (N = 1,072)

The Data Supplement describes the treatment received or prescribed treatment plan. Of the participants screened who were found to be positive with VIA and SEVIA, 80 received cryotherapy (7.5%), and 35 were referred for LEEP (3.3%); others were referred for biopsy or other consultation or treatment.

Results on the basis of HIV status are also reported in the Data Supplement. When disaggregated by HIV status, among HIV positive women, VIA-positive and SEVIA-positive results were almost identical, with VIA positivity rate was 14.9% and SEVIA positivity rate was 14.4%. A total of 89% (n = 8 of 9) of HIV-positive women who were VIA positive had lesions eligible for cryotherapy and were treated; eight VIA-positive women had large lesions and were referred for LEEP. A total of 99% (n = 72 of 73) of HIV-negative women who were VIA positive were treated with cryotherapy, with 27 women referred for LEEP.

**Fig 2:**
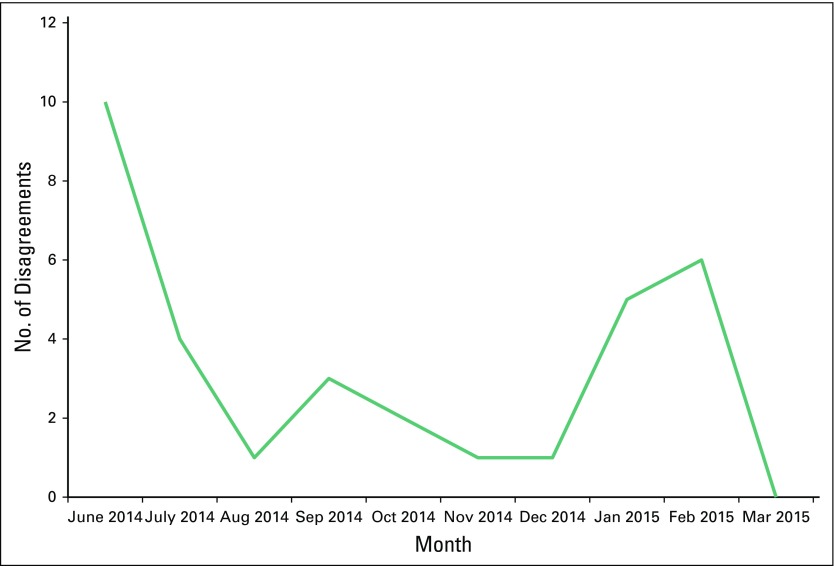
Disagreement between health care workers and experts by month through study period.

Figure 2 describes the level of agreement between CCS providers and expert reviewers across each month of the study. Initially, there was disagreement between CCS providers and expert reviewers of approximately 10%. This disagreement was reduced to less than 3% after the first month of the study and remained close to that level for most of the remainder of the study.

## DISCUSSION

This study was designed to evaluate the feasibility and effectiveness of using a smartphone camera to enhance VIA by obtaining digital photographic images of the cervix (cervicography) and sharing those images over a closed user group though text messaging on the mobile network so that experts can provide continuous real-time mentorship to CCS providers. This study also supports the WHO mandate that strengthening of task shifting and task sharing in CCS programs from physicians to nonphysician health workers through innovative approaches holds promise for the maintenance of a qualified and expanding health workforce to further decrease deaths resulting from cervical cancer in low-income countries.^17^

This study was not designed to determine if digital cervicography (traditional or smartphone based) would improve sensitivity of VIA, because this has already been established in the literature.^9,18,19^ This study was implemented with the knowledge that VIA programs, despite their relatively low cost and proven ability to detect cervical cancer in low-resource settings,^20^ continue to face challenges of providing effective quality assurance through maintenance of CCS provider skills and efficient and cost-effective methods to train and retrain CCS providers when needed. SEVIA has the ability to provide additional visual enhancement of VIA, but more importantly, when combined with mobile connectivity, it provides an excellent platform to strengthen and maintain VIA skills and expertise when combined with a well-structured and pragmatic mentorship program.

The use of smartphones to enhance VIA in almost any setting where there is a mobile network also provides the capability to easily archive and share images over time for training and mentorship as well as future review for quality assurance. In addition, it also allows for a permanent medical record (ie, an image) to strengthen referral procedures and follow-up, which are typically weak in many low-resource settings, where women may access screening but cannot access referral services for biopsy or treatment of higher-grade lesions.^11^ Furthermore, once diagnosed with late-stage cervical cancer, women in Tanzania face significant barriers to access to treatment, because Tanzania has only one comprehensive cancer center for a population of 48 million—the Ocean Road Cancer Institute, located in Dar es Salaam—which highlights the importance and necessity for quality CCS programs for early detection.

Our methodology demonstrated that CCS providers already trained in CCS rapidly developed the skills to use the smartphone for image collection and to perform high-quality VIA and SEVIA within a few weeks, such that 97% agreement was seen between trainees and experts by the end of the first month. The feasibility of this method for training and maintenance of skills was also strengthened by the minimal requirement for equipment and infrastructure (ie, mobile network, smartphone, solar-powered telephone charger, and mobile cryotherapy equipment that requires no electricity) in any setting, including mobile screening clinics. This allowed the screening teams to become mobile and reach women at risk in rural areas where screening was unavailable.

In addition, we found several unexpected outcomes, including the positive impact of the ability of a health worker to discuss a woman’s cervical cancer risk and show her an image of her cervix through the use of a smartphone camera. Our qualitative analysis (data not shown) suggests that this patient–provider engagement provided an important opportunity for health workers to empower women to learn about and seek additional information regarding their health, which for some is traditionally a taboo subject. Although we used locally available expert reviewers, this study highlighted several strengths that could be tested and implemented on a large scale within current VIA programs in Tanzania and other low-income countries with high cervical cancer prevalence. Quality assurance was built in and immediate in our protocol. Our program strengthened the goal of task shifting and sharing of VIA and SEVIA skills to CCS providers through the formation of teams of expert peer educators with ample VIA and SEVIA experience to mentor new trainees.

This study had some limitations. Screening accuracy of the CCS providers was evaluated by two expert reviewers who were carrying out their day-to-day jobs while providing timely review and feedback on cervical images transmitted to them by text message. For feasibility and pragmatism, we assumed that the expert reviewers’ assessment of the images was the gold standard. These expert reviewers had many years of experience (combined experience > 10 years) performing VIA and traditional digital cervicography. We did not perform an additional external expert review of the images, because one of the purposes of the study was to evaluate the feasibility of the program through use and impact of locally available skilled mentorship.

In conclusion, CCS with VIA enhanced by a SEVIA method and real-time ongoing expert mentorship through text messaging of images is both feasible and effective in a low-income country such as Tanzania. This study holds promise for further widespread adoption of high-quality CCS and treatment programs in low-income countries.
